# Dual RNA-seq reveals viral infections in asthmatic children without respiratory illness which are associated with changes in the airway transcriptome

**DOI:** 10.1186/s13059-016-1140-8

**Published:** 2017-01-19

**Authors:** Agata Wesolowska-Andersen, Jamie L. Everman, Rebecca Davidson, Cydney Rios, Rachelle Herrin, Celeste Eng, William J. Janssen, Andrew H. Liu, Sam S. Oh, Rajesh Kumar, Tasha E. Fingerlin, Jose Rodriguez-Santana, Esteban G. Burchard, Max A. Seibold

**Affiliations:** 1 Center for Genes, Environment, and Health, National Jewish Health, Denver, CO USA; 2 Department of Medicine, University of California, San Francisco, CA USA; 3 Department of Medicine, National Jewish Health, Denver, CO USA; 4 Department of Pediatrics, National Jewish Health, 1400 Jackson St, Denver, CO 80206 USA; 5 Children’s Hospital Colorado and University of Colorado School of Medicine, Aurora, CO USA; 6 Department of Pediatrics, The Ann and Robert H. Lurie Children’s Hospital of Chicago, Northwestern University Feinberg School of Medicine, Chicago, IL USA; 7 Department of Biomedical Research, National Jewish Health, Denver, CO USA; 8 Centro de Neumologia Pediatrica, San Juan, Puerto Rico; 9 Department of Bioengineering and Therapeutic Sciences, University of California, San Francisco, CA USA; 10 Division of Pulmonary Sciences and Critical Care Medicine, University of Colorado School of Medicine, Aurora, CO USA

**Keywords:** Asymptomatic, Virus, Infection, Transcriptome, Airway epithelium, RNA-seq, Asthma, Children, Host response, Host-virus interactions

## Abstract

**Background:**

Respiratory illness caused by viral infection is associated with the development and exacerbation of childhood asthma. Little is known about the effects of respiratory viral infections in the absence of illness. Using quantitative PCR (qPCR) for common respiratory viruses and for two genes known to be highly upregulated in viral infections (*CCL8*/*CXCL11*), we screened 92 asthmatic and 69 healthy children without illness for respiratory virus infections.

**Results:**

We found 21 viral qPCR-positive and 2 suspected virus-infected subjects with high expression of *CCL8/CXCL11*. We applied a dual RNA-seq workflow to these subjects, together with 25 viral qPCR-negative subjects, to compare qPCR with sequencing-based virus detection and to generate the airway transcriptome for analysis. RNA-seq virus detection achieved 86% sensitivity when compared to qPCR-based screening. We detected additional respiratory viruses in the two *CCL8/CXCL11*-high subjects and in two of the qPCR-negative subjects. Viral read counts varied widely and were used to stratify subjects into Virus-High and Virus-Low groups. Examination of the host airway transcriptome found that the Virus-High group was characterized by immune cell airway infiltration, downregulation of cilia genes, and dampening of type 2 inflammation. Even the Virus-Low group was differentiated from the No-Virus group by 100 genes, some involved in eIF2 signaling.

**Conclusions:**

Respiratory virus infection without illness is not innocuous but may determine the airway function of these subjects by driving immune cell airway infiltration, cellular remodeling, and alteration of asthmogenic gene expression.

**Electronic supplementary material:**

The online version of this article (doi:10.1186/s13059-016-1140-8) contains supplementary material, which is available to authorized users.

## Background

The detection of viruses from human biological samples and the determination of their influence on the host cell transcriptome are paramount for understanding complex human diseases that are initiated or exacerbated by virus infections and their sequelae. Even virus detection alone has proven challenging for current methods. First, PCR-based viral amplicon detection strategies are susceptible to cross-reactivity, lack strain specificity, and are inefficient for broad screening due to the lack of a common gene shared across virus species [1–3]. Solution-based capture methods followed by fluorescent detection or sequencing have generated quality data but can be confounded by sequence differences between viral species/strains and can only detect viruses for which the capture panel is designed [4, 5]. A third method involves the physical isolation of virions from clinical samples followed by sequencing to determine viral species present [6–9]. However, physical separation methods (e.g., ultra-centrifugation and ultra-filtration) are cumbersome to apply to multiple samples, and the following sequence-based detection may be unsuccessful when the sample viral load is low.

Another strategy is to perform shotgun sequencing of nucleic acids isolated from the entire, unseparated clinical sample (i.e., metagenomics). This method has been previously applied to total nucleic acids (RNA and DNA) extracted from clinical samples for viral detection [10–13]. Although metagenomic sequencing of total nucleic acids can detect viruses, its feasibility is highly dependent on the level of virus present in the sample and the amount of host cell nucleic acid present, which can in turn demand high levels of sequence depth for virus detection. Moreover, since the sequencing library contains reads from host DNA, the host transcriptome cannot be analyzed. An alternative is to isolate and specifically sequence RNA isolated from a clinical sample. The sequencing reads generated from this RNA sample can then be mapped to the human genome and analyzed to establish the host transcriptome profile, and unmapped reads can be queried against viral databases to establish the presence of both RNA and DNA viruses present in the sample through their transcriptional activity. For viruses with RNA genomes (the majority of respiratory viruses), the viral reads detected with this method can also originate from the virus genome in addition to active viral transcription. Total viral read counts generated, as a reflection of both infection level and transcriptional activity, can be tested for association with the host transcriptome data from the same sample. For multi-transcript viruses the read counts for specific viral transcripts could also be related to host transcript levels. This dual purposing of a single sequencing library is not only efficient, but powerful in that a level of internal control is achieved by use of a common sample, library, and sequencing reaction to generate both host and virus data. Such dual RNA-seq approaches have recently been applied to interrogate host-viral interactions both in vitro [14] and in vivo [15, 16], aiding greatly in the deconvolution of viral-driven disease mechanisms. In particular, the work of Perez-Losada et al., 2015 [16] established the first dual RNA-seq study in the context of asthma, focusing on differences in the host-bacterial microbiome interactions between asthmatic and healthy children.

Upper respiratory tract infections are among the most prevalent of all viral infections in humans and render a dramatic cost on the health care system in terms of lost economic productivity and general well-being. Beyond their acute effects, respiratory virus infections are strongly associated with the development, symptoms, and exacerbation of asthma [17, 18]. For example, an early-life infection with respiratory syncytial virus (RSV), which induces respiratory bronchiolitis, is associated with a sixfold increase in asthma risk by age 6 years [19]. Early-life rhinovirus-associated wheezing has an even stronger association with subsequent asthma by age 6 and 13 years [20, 21]. Rhinovirus infections are also associated with >80% of childhood asthma exacerbations [22], and recently rhinovirus detection was associated with increased day-to-day asthma symptoms throughout the year while, at times, dissociated from nasal symptoms and exacerbations [23, 24]. Despite the dramatic increase in risk associated with respiratory tract infections, it is also clear that most people affected by respiratory viruses are resilient to development of asthma or a significant exacerbation. These facts suggest a complex interaction between the host and virus, which is important for determining risk. This interaction first occurs at the level of the nasal airway epithelium, which is the primary site of infection and replication for respiratory viruses. The response of the airway epithelium to viral infection is complex and involves both the mucociliary epithelial and interdigitating immune cells that form the mucosal surface. This intricate cellular milieu is difficult to recreate for in vitro experimental infection studies; therefore, novel methods are needed to capture this biology in the in vivo setting.

In this study, we detail methods for the detection of common respiratory viruses in asthmatic and healthy children using polyA-selected whole transcriptome sequencing. Using these efficient methods, we found that a large proportion of children without respiratory illness were carrying pathogenic and transcriptionally active respiratory viruses. Virus detection using a dual RNA-seq workflow resulted in 86% sensitivity when compared to quantitative PCR (qPCR)-based screening. Additionally, we detected respiratory viruses not targeted by viral qPCR primers in two subjects with high expression of viral response genes, demonstrating the versatility of this method. Despite absence of respiratory illness, viral carriers exhibited a dramatically altered nasal epithelial transcriptome, reflective of active viral infection. Virus levels determined by RNA-seq correlated strongly with host airway viral response gene expression levels. Even virus levels at the limits of detection by qPCR and RNA sequencing had a discernible effect on the host airway epithelial transcriptome. We detail modules of activated gene expression in viral carriers reflective of epithelial cell subtypes, as well as immune cell types. We also report modules of asthmogenic genes that are strongly associated with viral infection. We conclude that viral infection, even in the absence of observable illness, induces epithelial responses in children with and without asthma.

## Results

### Quantitative PCR screening for respiratory virus infections in 161 subjects without respiratory illness

**Fig. 1:**
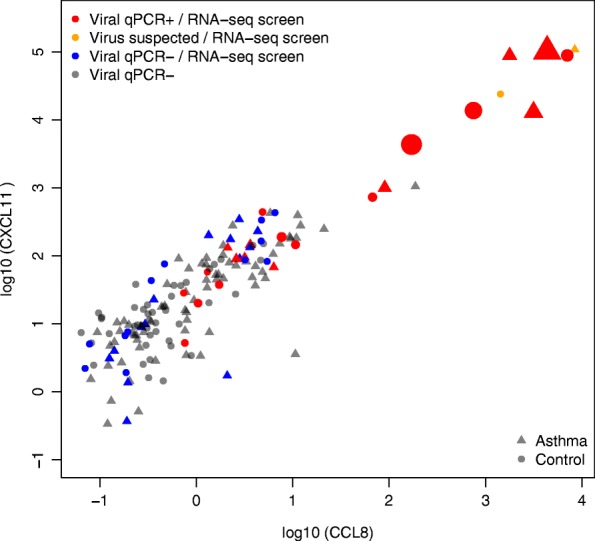
Viral detection and viral biomarker qPCR results with RNA-seq subject selection. Expression of *CCL8* and *CXCL11* in 161 subjects was screened by qPCR. Respiratory virus﻿es﻿ were detected by qPCR i﻿n the samples colored red.; all of those subjects were then sequenced with RNA-seq. Samples colored in *orange* show the two subjects with suspected viral infection, despite viral qPCR negative results, based on the expression of *CCL8* and *CXCL11*; both of those subjects were selected for RNA sequencing. Samples colored in *blue* represent subjects with no respiratory viruses identified with qPCR and selected for the RNA-seq part of the study. The remaining samples (in *gray*) represent subjects with no respiratory viruses identified with qPCR and not selected for the RNA-seq part of the study. Shape of the points represents asthma status, while the size corresponds to 2^(−Ct)^ value for virus detection by qPCR

We first used qPCR assays for six common respiratory virus species to identify which of the 161 Genes-environments and Admixture in Latino Americans II (GALA II) cohort subjects (92 asthmatics and 69 healthy controls) were infected with a respiratory virus, despite the absence of respiratory illness (Additional file 1: Table S1, Additional file 2: Figure S1). Specifically, the qPCR assays tested for human rhinovirus (HRV), respiratory syncytial virus (RSV), human metapneumovirus (HMPV), human parainfluenza virus 1 (HPIV1), human parainfluenza virus 2 (HPIV2), and human parainfluenza virus 3 (HPIV3) [25]. We identified 21 subjects harboring respiratory viruses (9 asthmatics, 12 controls): 14 HRV, 4 RSV, 3 HPIV1, and 1 HMPV (1 subject was qPCR-positive for 2 viruses) (Table 1). To screen for additional subjects carrying other viral species, we performed a qPCR assay on the subject RNA for expression of two putative viral biomarker genes. These genes, *CCL8* and *CXCL11*, were previously reported as among the most upregulated during the peak stage of acute respiratory illness [26]. Our qPCR screen found that most subjects exhibited low expression for both of these genes, and that subjects with high expression of these genes were mostly qPCR positive for viruses (Fig. 1, Table 1). Moreover, among the virus-positive subjects, we observed high correlation between both the *CCL8* and *CXCL11* gene expression and virus levels (ρ_*CCL8*_ = 0.84, ρ_*CXCL11*_ = 0.80). Two subjects that were ranked in the top fifth percentile of expression for both biomarker genes were negative for virus by qPCR, and were therefore suspected to be carriers of viral species not covered by the qPCR panel (Fig. 1). There was no significant difference in viral carriage rate observed between asthmatics and healthy controls.

**Table 1:** Amplication and mapping metrics for samples with respiratory viruses detected by RNA-seq and/or by qPCR. Detected virus coverage was scaled by the total number of raw reads divided by 10e6. If the closest reference differed between the multiple contigs for a sample, then the most frequent closest reference sequence was reported. In all cases the closest reference for all contigs of a sample were of the same viral species (i.e., no dual infections were detected)

Subject	qPCR				RNA-seq mapping metrics			Detected virus				
	Virus		Gene		Total reads	Resp. virus reads	% Viral reads	Species	Strain/serotype	Closest reference (NCBI acc)	Virus mapped to NCBI reference	Coverage (scaled)
	Species	Ct	*CCL8*	*CXCL11*								
Asthma-1	None	NA	8426.9	109,137.3	10,720,551	164,870	1.54	HPIV	4a	KF483663.1	98.9%	1271.6x
Control-1	HRV	29.36	7.7	190.8	9,818,450	74,935	0.763	HEV	C105	JX393302.1	92.8%	1259.7x
Asthma-2	RSV	20.05	4356.0	103,107.1	11,597,132	58,263	0.502	RSV	A	KJ672478.1	99.8%	473.5x
Control-2	HPIV1	24.37	749.7	13,819.3	14,029,017	26,045	0.186	HPIV	1	KF687314.1	99.8%	172.2x
Control-3	HRV	24.19	170.7	4377.0	10,591,814	4081	0.039	HRV	A55	DQ473511.1	80.8%	66.9x
Asthma-3	HPIV1	25.29	3148.7	13,129.6	9,738,089	6648	0.068	HPIV	1	KF530212.1	99.8%	64.4x
Control-4	HRV	25.86	7042.3	89,477.7	16,450,743	2878	0.017	HRV	A59	JN541266.1	99.3%	37.9x
Asthma-4	HRV	38.15	6.4	68.5	15,103,156	829	0.005	HRV	C	JF317015.1	99.9%	11.7x
Control-5	None	NA	1430.1	24,191.0	14,863,334	2671	0.018	Influenza	B	CY171998.1	5.7%	7.8x
Control-6	HRV	30.24	67.3	734.7	9,732,129	203	0.002	HRV	A59	JN541266.1	99.5%	4.5x
Asthma-5	HRV	29.17	90.1	1010.9	8,269,118	156	0.002	HRV	A100	FJ445175.1	91.0%	3.6x
Control-7	None	NA	4.7	166.0	10,579,380	780	0.007	HCoV	OC43	KF530099.1	95.0%	3.2x
Asthma-6	HRV	28.60	1780.1	89,078.1	9,944,802	97	0.001	HRV	A55	DQ473511.1	85.6%	1.7x
Control-8	HMPV	30.78	10.7	146.7	9,978,447	107	0.001	HMPV	A2	GQ153651.1	99.1%	1.2x
Control-9	HRV	40.98	0.7	28.6	14,107,522	76	0.0005	HRV	C	JF317015.1	100%	1.2x
Control-10	HRV	44.08	1.3	58.1	14,005,041	39	0.0003	HEV	CY135	KF322116.1	15.4%	0.7x
Control-11	HRV	33.35	1.0	20.2	15,787,336	35	0.0002	RSV	A	EF155421.1	100%	0.5x
Control-12	None	NA	0.5	76.5	10,880,596	24	0.0002	RSV	A	KJ672484.1	100%	0.2x
Asthma-7	HRV	37.60	2.6	90.8	16,883,056	9	5.33e-05	HCoV	NA*	JX504050.1	66.7%	NA*
Asthma-8	RSV	35.95	3.6	145.9	15,782,330	7	4.44e-05	RSV	NA*	KJ672484.1	85.7%	NA*
Asthma-9	HRV	42.14	2.1	132.1	13,910,896	5	3.59e-05	HRV	NA*	KC342104.1	40%	NA*
Control-13	HRV	33.51	1.7	37.8	17,449,199	4	2.29e-05	HRV	NA*	AB904651.1	25%	NA*
Control-14	HRV	37.76	0.8	5.3	14,074,760	0	0	NA	NA	NA	NA	NA
Asthma-10	RSV/HPIV1	40.04/37.25	3.2	95.6	15,013,585	0	0	NA	NA	NA	NA	NA
Control-15	RSV	37.8	4.9	443.7	17,496,674	0	0	NA	NA	NA	NA	NA

*Too few reads to perform assembly into contigs

### Respiratory virus detection in a host-dominated transcriptome

**Fig. 2:**
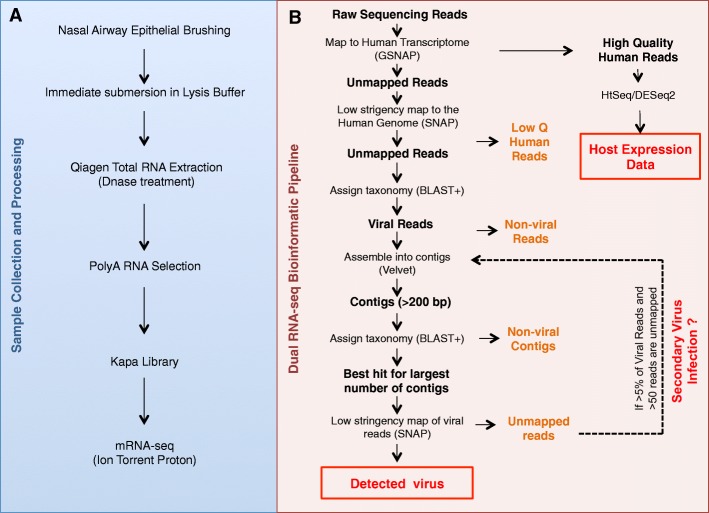
Experimental design and methods for dual RNA-seq of nasal airway epithelial brushings. **a** Workflow for sample collection, processing, and library preparation. **b** Dual RNA-seq bioinformatic pipeline

**Fig. 3:**
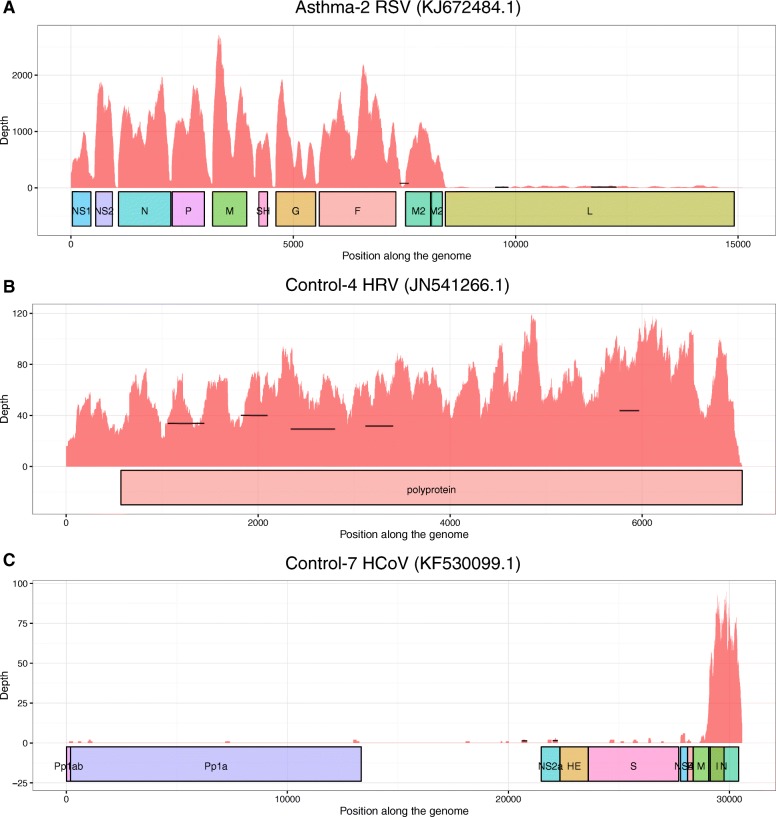
Representative genome coverage plots of respiratory viruses detected in nasal transcriptome samples. The coverage depth values were obtained by direct mapping of all the viral reads to the detected viral sequence with SNAP. *Horizontal lines* represent the position of the assembled contigs obtained with Velvet, plotted at their reported depth. **a** Multi-transcript virus genome: respiratory syncytial virus detected in transcriptome of Asthma-2. **b** Single polyprotein transcript virus genome: human rhinovirus detected in transcriptome of Control-4. **c** Near monogenic viral genome coverage for human coronavirus detected in transcriptome of Control-7

We next generated polyA-selected whole transcriptome RNA-seq data on the 21 qPCR virus-positive subjects, 2 *CCL8*/*CXCL11*-high but viral qPCR-negative subjects, and 25 randomly selected viral qPCR-negative subjects. We applied an adapted Kapa mRNA-seq protocol to generate the transcriptome libraries from these 48 samples (Fig. 2a). The samples were sequenced to a mean read depth of 1.26 × 10^7^ ± 2.71 × 10^6^ using the Ion Torrent Proton sequencer. This sequencing depth was chosen based on what was previously determined to yield informative airway epithelial transcriptome data [27]. We analyzed these data using a pipeline of publicly available bioinformatic tools to detect and quantify respiratory virus reads in the samples, in addition to host transcriptome generation (Fig. 2b). Across samples we found that 3.5–7.0% of reads failed to map with high stringency to the human genome. We therefore attempted a second low-stringency alignment of these unmapped reads to the human genome using a different aligner, which resulted in an average of 87.5% (±6.4%) of these additional reads being mapped. To identify reads of viral origin, we performed a Basic Local Alignment Search Tool (BLAST) search of unmapped reads against the National Center for Biotechnology Information (NCBI) Nucleotide database. We found respiratory virus reads in 22 of 48 subjects sequenced. The viral reads were assembled into longer contigs to enable more accurate taxonomic assignment of the read source. All reads not mapping to the human genome for these samples were subsequently mapped to their respective contig-matching virus genome. Only a single virus species was detected in each sample, and the number of viral reads varied significantly, from as little as 4 reads (2.29 × 10^−5^% of the total reads) to 164,870 reads (1.54% of the total reads) generated per sample. Viral genome read depth plots were generated for all virus-infected samples with >20 viral reads (Fig. 3, Additional file 2: Figure S2). For all but one of these viral carriers, we observed coverage spread throughout the length of the detected viral genome (Fig. 3a, b), providing us with confidence that the virus-matching reads were not sequencing or mapping artifacts. In the sample with uneven viral read distribution (Fig. 3c), Control-7, nearly all HCoV reads mapped to the virus nucleocapsid gene. Interestingly, viral genome read depth plots for multi-transcript viruses, such as parainfluenza and RSV, revealed breaks in the read depth across the genome aligned with viral transcripts, indicating that the reads originated from viral transcription and not simply from viral RNA genomes (Fig. 3a).

### RNA-seq versus qPCR virus detection

In total, respiratory virus reads were detected in 18 of 21 qPCR virus-positive samples, resulting in a sensitivity of 86% using RNA-seq at this depth (Table 1). The three samples with viruses not detected by RNA-seq all had viral qPCR Ct values at the edge of detection (Ct > 37, Table 1). The respiratory viruses detected by RNA-seq matched the viruses identified with qPCR in 15 out of 18 cases. In the three discordant cases, the viral species identified with qPCR was HRV; however, RNA-seq-based screening identified human enterovirus for two of the subjects and human coronavirus for the third. Enterovirus and HRV are part of the same viral genus and share high sequence similarity [28], which is likely why the HRV primers gave a positive signal in the enterovirus-infected samples. Careful examination of the third sample confirmed all reads mapped to coronavirus, confirming these results. Although HRV reads were not detected in this sample, the level of HRV infection detected in this sample was very low (Ct >37). We found that 23 of the 25 qPCR virus-negative subjects were RNA-seq-negative for respiratory viruses. One of the RNA-seq virus-positive samples, Control-12, exhibited 24 RSV reads. The other sample, Control-7, exhibited 780 reads matching an unscreened human coronavirus (HCoV).

Examining the two qPCR-negative, but suspected virus carriers based on nasal airway epithelial viral biomarker expression, we detected human parainfluenza virus 4a (HPIV-4a) in one sample and influenzavirus B in the other sample. Neither viral species was tested in our qPCR assays. Examining all samples with virus detected by both methods, there was a strong positive correlation between qPCR viral signal and the proportion of viral reads in RNA-seq data (ρ = 0.75, *P* = 3 × 10^−3^). Therefore, RNA-seq viral detection at this low read depth is highly quantitative, is nearly as sensitive as qPCR viral assay, and has likely unlimited breadth of detection. Examining all subjects either qPCR or RNA-seq positive for virus, we observed a skew in the frequency of virus-infected subjects by season (*P* = 0.042). This skew was driven by a significant decrease in viral infection frequency in summer (virus positive 4%, virus negative 23%, *P* = 0.029) and a trend toward increased infection in the fall (virus positive 24%, virus negative 10%, *P* = 0.092).

### Respiratory virus infection without respiratory illness drives a dramatic shift in the host transcriptome

**Fig. 4:**
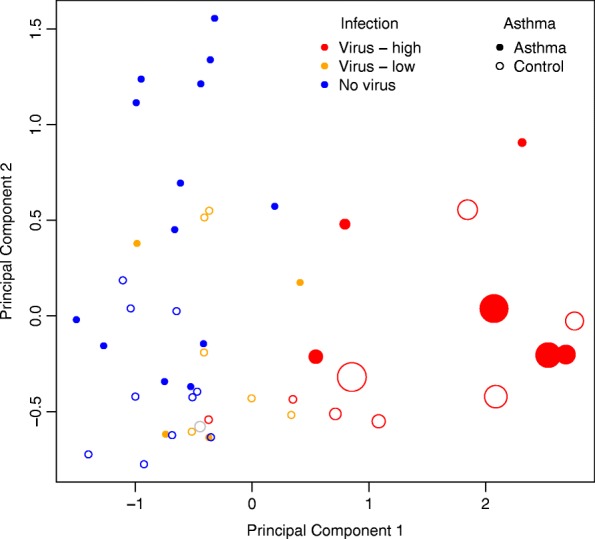
Multidimensional scaling plot of host airway brushing gene expression for virus-infected and non-infected samples. Data shown for Ion Torrent Proton sequenced nasal transcriptome samples among 14 Virus-High subjects (*red*), 10 Virus-Low subjects (*orange*), and 23 No-Virus subjects (*blue*). Note: the Control-7 sample, which exhibited monogenic viral genome coverage, is shown in *gray*. Asthmatics are marked with *filled circles*, while healthy controls are plotted with *empty circles*. Circle sizes correspond to coverage depth of the viral sequence detected with RNA-seq

We then examined whether the host airway epithelial transcriptome generated from the same sequencing libraries was reflective of viral infection status. Due to the wide variation in the number of viral reads detected, we divided the viral carriers into two groups based on RNA-seq virus detection: (1) Virus-High subjects: with viral genome coverage of > =1x (*n* = 14) and (2) Virus-Low: remaining subjects harboring a respiratory virus (*n* = 10) (Table 1). Given the near monogenic read pileup for subject Control-7, we did not include this subject in any of the downstream analyses. The 23 subjects with no respiratory virus detected by either qPCR or RNA-seq comprised the No-Virus group. We first performed multidimensional scaling (MDS) of the airway epithelial transcriptome data (Fig. 4, Additional file 1: Table S2). The first dimension generated from this analysis accounted for 36.9% of total transcriptome variance. It completely separated the Virus-High subjects from No-Virus subjects. The Virus-Low subjects clustered in between the Virus-High and No-Virus subjects; however, they were not completely distinct from the No-Virus subjects. Dimension 1 values correlated with observed viral depth (ρ = 0.47). We therefore found the stratification of samples by high versus low viral genome coverage depth to be strongly associated with the intensity of the airway expression response. To identify specific genes associated with virus carriage without illness, we performed single gene differential expression analysis between the Virus-High and No-Virus samples. We found 8126 differentially expressed genes (false discovery rate, FDR 5%), including 4061 upregulated and 4065 downregulated genes (Additional file 1: Table S3). Among the top genes we observed host virus response genes (*RAD2*, *OASL*, etc.) and many interferon-induced genes (*ISG15*, *IFIT2*, *IFIT1*, *IFITM3*, *IFIT3*, etc.). Moreover, Gene Ontology (GO) analysis of the top differentially expressed genes (FDR-corrected *P* value ≤0.01 and log2 fold change absolute value >1, genes = 2148) resulted in hits for defense response to virus (*P* = 4.5 × 10^−23^) and the virus responsive, interferon-gamma-mediated signaling pathway (*P* = 2.1 × 10^−21^) (Additional file 1: Table S4).

We next examined whether genes previously shown to be upregulated in acute viral infection of the nasal airway are discriminatory for infected subjects without illness. We found 55 of the 56 genes previously reported as upregulated during the peak stage of acute viral respiratory illness were upregulated in our Virus-High subjects (all 55 genes FDR corrected differential expression *P* values <1.0 × 10^−4^ and log2 expression fold changes >2) [26].

Secondly, we determined the in vitro primary airway epithelial transcriptional response to acute respiratory virus infection to draw comparisons to the transcriptional signature exhibited in vivo among the Virus-High subjects without illness. To accomplish this, we performed whole transcriptome paired differential expression analysis of HRV-A16-infected and mock-infected mucociliary differentiated airway epithelial cell cultures from three donors (Additional file 1: Table S5). We identified 493 airway epithelial genes significantly differentially expressed between the HRV-A16- and mock-infected cultures (1% FDR and absolute log2 fold change in expression >2, Additional file 1: Table S6). We found 92.8% of these genes were differentially expressed in our Virus-High subjects compared to No-Virus subjects. Moreover, the fold changes in expression between our in vitro and in vivo datasets were highly correlated (Additional file 2: Figure S3, ρ = 0.94, *P* < 2.2 × 10^−16^). These results indicate that respiratory virus infection of the airway epithelium without clinical illness can mediate epithelial expression changes characteristic of active acute infection.

### Low-level viral infection without illness is associated with airway epithelial viral responses

**Fig. 5:**
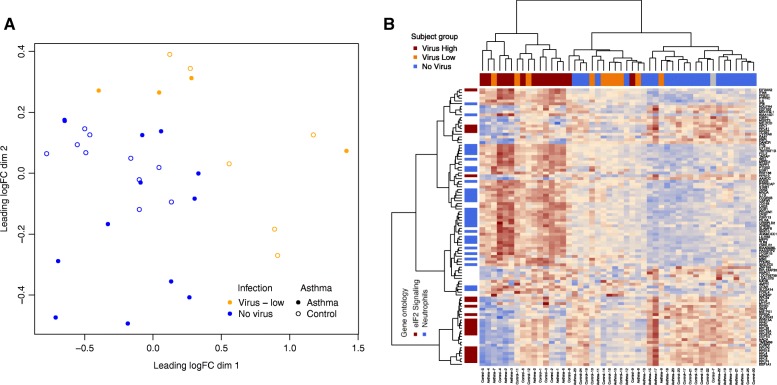
**a** MDS plot on 100 differentially expressed genes between Virus-Low and No-Virus groups. **b** Hierarchical clustering of study subjects on 100 differentially expressed genes between Virus-Low and No-Virus groups. Column side colors indicate virus grouping (*dark red* = Virus-High, *orange* = Virus-Low, *blue* = No-Virus), while row side colors indicate gene functional categories (*dark red* = eIF2 signaling pathway, *blue* = neutrophil enriched genes based on Gene Set Enrichment Analysis (*GSEA*) of these genes in the neutrophil versus other immune cell types distribution)

We next investigated whether any of the host viral responses seen in Virus-High subjects were also present in the Virus-Low individuals. Performing transcriptome-wide single gene differential expression, we identified 100 differentially expressed genes (FDR 5%) between the Virus-Low and No-Virus subjects (Additional file 1: Table S7). We found that 80 of those genes were also significantly differentially expressed between the Virus-High and No-Virus subjects. We found that 42 of the 100 genes were downregulated in virus-infected samples, and were significantly enriched in genes from the eIF2 signaling pathway (*P* = 1.67 × 10^−30^, Ingenuity Pathway Analysis (IPA)). Closer investigation of those genes revealed downregulation of the ribosomal subunit genes, which predicted activation of *EIF2AK2*. The *EIF2AK2* gene encodes for a kinase which is activated by virus infection to inhibit expression of translational machinery, as a host viral defense mechanism [29]. Importantly, *EIF2AK2* expression was among the 58 significantly upregulated genes in the Virus-Low subjects. The upregulated genes showed a significant enrichment in non-activated (*P* = 3.89 × 10^−09^) and activated neutrophil (*P* = 1.82 × 10^−07^) signatures, as well as an activated macrophage signature (*P* = 3.20 × 10^−02^). These 100 genes separated the Virus-Low subjects from No-Virus subjects in an MDS analysis (Fig. 5a). Hierarchical clustering of all 48 subjects from this study using these genes demonstrated even higher expression levels in the Virus-High group (Fig. 5b). In fact, the collapsed expression of these genes is highly correlated with the read depth of respiratory virus carried (ρ = 0.73).

### Network analysis of epithelial response to viral infection without illness

In an effort to better understand the mechanisms activated among virus-infected samples, we applied weighted gene co-expression network analysis (WGCNA) [30] to the 8126 differentially expressed genes from the Virus-High to No-Virus comparison. WGCNA utilizes a correlation network approach to identify modules of highly correlated genes, likely representing a common biological process or pathway. WGCNA analysis detected nine distinct network modules (Table 2, Additional file 2: Figure S4, Additional file 1: Table S8) comprising 3378 of the differentially expressed genes. Modules were assigned arbitrary color names by the WGCNA package, and expression values of genes within each module were summarized by a single module eigengene value for each subject/module.

**Table 2:** WGCNA module summary and functional enrichments

Module (gene number)	Hub genes	Viral Cor.*	Enrichment terms	Immune cell type enrichment
Pink (100)	SPI1	0.83	GO:0006952 defense response (*P*_adj_ = 6.17e-07)	Neutrophils (*P* = 7.46e-11)
	PLEK		GO:0006955 immune response (*P*_adj_ = 2.25e-06)	Act. neutrophils (*P* = 4.44e-09)
	HCK		IPA path: Fcγ receptor-mediated phagocytosis in macrophages and monocytes (*P* = 4.97e-10)	Eosinophils (*P* = 3.74e-04)
	FGR		IPA upstream regulator: IFNG (*P* = 4.77e-16)	Act. macrophages (*P* = 9.78e-03)
	TLR4		IPA function: leukocyte migration (increased) (*P* = 3.82e-22)	Act. eosinophils (*P* = 2.67e-02)
Blue (726)	SLAMF7	0.75	GO:0006955 immune response (*P*_adj_ = 5.31e-76)	Act. macrophages (*P* = 2.49e-16)
	SP100		GO:0006952 defense response (*P*_adj_ = 2.15e-29)	Dendritic cells (*P* = 3.01e-11)
	DTX3L		GO:0045321 leukocyte activation (*P*_adj_ = 3.79e-26)	Act. neutrophils (*P* = 3.78e-05)
	GIMAP4		IPA upstream regulator: IFNG (*P* = 1.66e-100)	Neutrophils (*P* = 1.99e-04)
	OAS3		IPA path: interferon signaling (*P* = 3.35e-22)	NK cells (*P* = 2.64e-02)
Black (117)	DCAKD	0.28	GO:0031982 ~ vesicle (*P*_adj_ = 1.38e-02)	Act. macrophages (*P* = 4.06e-03)
	GNA13		GO:0012505 endomembrane system (*P*_adj_ = 2.10e-02)	
	PICALM		IPA upstream regulator: CHEK2 (*P* = 6.49e-05)	
	CREB1		IPA path: ephrin receptor signaling (*P* = 3.26e-04)	
	LARP4		IPA function: viral infection (increased) (*P* = 1.02e-04)	
Magenta (85)	TNKS2	0.32	*No significant DAVID enrichments*	Act. macrophages (*P* = 4.57e-02)
	VCPIP1		IPA path: oxidative phosphorylation (*P* = 8.05e-05)	
	MIER1		IPA path: mitochondrial dysfunction (*P* = 6.51e-04)	
	CD2AP		IPA path: NF-κB signaling (*P* = 6.14e-03)	
	ARAP2			
Yellow (189)	CHST9	0.70	*No significant DAVID enrichments*	Act. neutrophils (*P* = 4.00e-04)
	ITGA5		IPA function: inflammatory response (*P* = 2.09e-11)	Act. macrophages (*P* = 9.78e-03)
	C8orf47		IPA function: quantity of leukocytes (*P* = 1.67e-10)	Neutrophils (*P* = 1.56e-02)
	OXSR1		IPA function: differentiation of cells (*P* = 1.74e-10)	Act. eosinophils (*P* = 1.83e-02)
	ITPRIPL2			
Green (179)	RPL10A	−0.46	GO:0006414 translational elongation (*P*_adj_ = 3.65e-44)	B cells (*P*_adj_ = 1.07e-07)
	RPL3		KEGG: hsa03010 ribosome (*P*_adj_ = 3.34e-37)	Th2 cells (*P*_adj_ = 8.30e-05)
	EEF2		GO:0006412 translation (*P*_adj_ = 7.72e-36)	T cells (*P*_adj_ = 2.33e-04)
	RPS14		IPA path: eIF2 signaling (*P* = 4.07e-47)	Mast cells (*P*_adj_ = 1.33e-03)
	RPL4			Th1 cells (*P*_adj_ = 2.42e-02)
Brown (203)	ELOVL5	−0.74	Type 2 inflammation (*P* = 3.30e-04)	*No significant immune cell enrichments*
	CDH26		*No significant DAVID enrichments*	
	ALOX15		IPA upstream regulator: IL13 (*P* = 2.77e-05)	
	FETUB			
	VWF			
Red (164)	CFL1	0.50	GO:0015629 actin cytoskeleton (*P*_adj_ = 2.41e-06)	Act. neutrophils (*P*_adj_ = 2.35e-02)
	CAP1		GO:0031252 cell leading edge (*P*_adj_ = 6.68e-05)	Act. macrophages (*P*_adj_ = 3.04e-02)
	VASP		IPA path: remodeling of epithelial adherens junctions (*P* = 2.42e-09)	
	DIAPH1		IPA upstream predictor: TGFB1 (*P* = 6.88e-09)	
	TMBIM1		IPA function: organization of cytoskeleton (increased) (*P* = 3.65e-15)	
Turquoise (1,615)	C6orf165	−0.43	GO:0005929 cilium (*P*_adj_ = 2.78e-30)	*No significant immune cell enrichments*
	ARMC2		GO:0015630 microtubule cytoskeleton (*P*_adj_ = 1.02e-23)	
	CAPSL		GO:0005930 axoneme (*P* = 3.01e-21)	
	EFCAB6		IPA upstream regulator: RFX3 (*P* = 1.74e-05)	
	ANKRD66		IPA function: formation of cilia (decreased) (*P* = 1.47e-43)	

*Cor = Spearman correlation of module eigengenes with depth of detected viral genomes (for virus high samples only)

#### Activated immune cell modules

Five of the detected modules (pink, blue, black, magenta, and yellow) had highly correlated expression patterns, which were characterized by high module eigengene expression values in Virus-High subjects and low values in the No-Virus samples, regardless of asthma status (Table 2). Enrichment analysis of the module member genes for Gene Ontology (GO) terms found that immune response, defense response, and leukocyte activation terms were enriched in pink and blue modules (Additional file 1: Table S9). We hypothesized that these modules represent a signature of immune cells infiltrating upper airways in response to a viral infection. To test this hypothesis, we compared the results of in vitro stimulation of epithelial cells with HRV with our in vivo data and identified a set of 199 genes highly upregulated in the in vivo samples but not in the in vitro experiment (i.e., representing genes that may be expressed by immune cell types that are not represented in the in vitro airway epithelial cell-restricted experiment; Additional file 2: Figure S3). Interestingly, we found *CCL8* among the 199 genes, suggesting that this gene is only expressed by the immune cells infiltrating the nasal airway. We found strong over-representation of these 199 in vivo infection-specific genes in the blue and pink modules by hypergeometric testing (blue: 121 genes, *P* = 3.10 × 10^−65^; pink: 28 genes, *P* = 2.11 × 10^−20^), with no over-representation of these genes found among the remaining expression modules. We then explored which activated leukocyte populations might be represented by these modules by examining enrichment of the module genes in the publicly available expression datasets of specific activated immune cell populations [31] (Additional file 1: Table S10). The blue module indicated strong activated macrophage and neutrophil signatures, as well as enrichment for a dendritic cell signature. The pink module was most strongly enriched for neutrophil genes, with a weaker but still significant enrichment for activated macrophage and eosinophil genes. Black, magenta, and yellow modules were all significantly enriched for the activated macrophage signatures, and the yellow module was also enriched for neutrophil and eosinophil signatures. Additionally, we examined cytospins from second brushings of several infected study subjects, which were collected at the same time as the first brushing used for RNA-seq. We observed both macrophages and neutrophils among the airway epithelial cells in these cytospins (Additional file 2: Figure S5).

#### Virus-Low group module

The green module was significantly enriched in ribosomal genes (*P* = 1.52 × 10^−33^) and genes involved in the eIF2 signaling pathway, and had a statistically significant overlap with the genes differentially expressed in the Virus-Low group (*P* = 3.23 × 10^−08^, Table 2, Additional file 1: Table S7). Green module eigengenes were downregulated in the virus carriers, including the Virus-Low group. Overall module expression was similarly downregulated in the Virus-High subjects.

#### Modules of asthma-associated genes

The remaining three modules were associated to varying degrees with asthma among non-viral carrying samples (Table 2). In fact, 59% of the 3378 virus-associated genes incorporated into modules from our network analysis were within these three asthma-associated modules. In particular, the brown module’s expression was much higher among non-infected asthma samples compared to non-infected healthy controls. Close examination of this module’s genes revealed a signature of type 2 airway epithelial inflammation. In fact, the biomarkers of type 2 asthmatic airway inflammation (*POSTN*, *CLCA1*, *CST1,* and *ALOX15*) [32, 33] were all present in this module. Ingenuity Pathway Analysis (IPA®, QIAGEN, Redwood City, CA, USA, https://www.qiagenbioinformatics.com/products/ingenuity-pathway-analysis/) predicted IL-13, the primary driver of Th2 inflammation [34], to be the main upstream regulator of this module. These genes and the overall module eigengene expression were significantly lower in virus-infected versus non-infected subjects. In contrast, the red module eigengene values were higher in non-infected asthmatics versus non-infected controls, but were upregulated with viral infection among both asthmatic and control subjects. The red module was enriched for genes that encode for the production of actin cytoskeleton and the remodeling of epithelial adherens junctions genes (Additional file 1: Table S9). Expression eigengene values for the turquoise module were lower among non-infected asthmatics versus non-infected controls, and were downregulated among virus carriers. The turquoise module was the largest module, comprising 1615 genes. Genes in this module were greatly enriched in both cilium and microtubule cytoskeleton GO categories, and decreased formation of cilia was among the enriched functions identified by IPA. This is supported by recent work observing significant downregulation of cilium genes in airway epithelial cultures in response to rhinovirus infection [35]. Lastly, we found that genes within this module were highly enriched for *RFX3* transcription factor binding sites. The RFX family of transcription factors is known to drive the expression of genes involved in motile cilia formation of metazoans [36].

## Discussion

Dual RNA sequencing of host and microbe has been proposed as a groundbreaking method to simultaneously detect/quantify microbes and reveal their influence on the host transcriptome [37]. To date, however, there are still few examples of this method applied to human biological samples [16, 38]. This method is especially needed for viral detection, due to the high sequence variability of viruses and their lack of a universal marker gene. We detail here a dual RNA-seq workflow for both the detection/quantification of respiratory viruses and generation of host transcriptome data from a single nasal respiratory epithelial brushing library. Our analysis confirms the common nature of respiratory virus carriage without illness in children and finds that this carriage, even at low levels, has a dramatic impact on respiratory mucosa function.

We suspect that several features of our study design aided in successful virus detection in these samples. First, our immediate lysis of the complete sample and application of the total lysate onto the RNA column without pre-processing steps allowed us to avoid cumbersome, potentially biased, separation methods that could have resulted in loss of viral RNA. Secondly, we took advantage of the fact that most respiratory viruses have polyadenylated RNA genomes and that their expressed transcripts are also polyadenylated [39–41]. We performed standard polyA enrichment of total RNA isolated from the nasal samples and were therefore able to enrich libraries for respiratory virus sequences in the context of a standard library preparation for human whole transcriptome sequencing. Importantly, we find that our modest transcriptome sequencing depth (~1.2 × 10^7^/sample) was sufficient to detect virus infection at species and even strain level among subjects without illness.

Detection of viruses in asymptomatic children has been previously reported by multiple PCR studies, which detected viruses in 33–52% of asymptomatic subjects using nasopharyngeal swabs and washings [23, 24, 42–45]. The observed prevalence in these studies is higher than the rate observed in our samples (15.5%, detected by RNA-seq or qPCR). Furthermore, unlike previous studies, we did not detect any dual viral infections. We believe that this may be explained by sample type (brushings versus washes). We hypothesize that washes may sample a larger surface area of the nasal passages, and represent a sort of “environmental sampling” of nasal airway. In contrast, the brushings result in a point sampling of airway cells and may reflect viral infection of airway epithelial cells, rather than viral material passively inhaled and deposited on the nasal epithelium surface. Although we observed close correlation in viral qPCR and RNA-seq detection results, other studies have found discrepancies in PCR-based and other detection methods. For example, one study found that PCR-based testing of samples resulted in asymptomatic carriage rates of 41.7% versus 4.4% using conventional methods (immunochromatography, direct fluorescent antibody techniques, etc.) in the same samples [43]. In contrast to PCR-based detection of short viral sequences, with RNA-seq-based detection we are able to cover large portions of the virus genomes, as well as determine the host epithelial responses, which lends high credibility to the metatranscriptomics method. In particular, the virus genome read pileups with breaks between viral transcripts, for the multi-transcript virus genomes, indicate that this method detects transcriptionally active viruses. The theoretically unlimited breadth of detection for metatranscriptomic sequencing was apparent with our detection of respiratory virus species not targeted with viral qPCR primers, including HPIV strain 4a and influenzavirus B. Additionally, in three cases, the viral qPCR method indicated that the respiratory virus present in the sample was human rhinovirus; however, closer examination of all the viral sequencing reads revealed that the actual virus present was human enterovirus for two of the subjects and human coronavirus in the third case. We take the RNA-seq result to be correct since it is based on detection of a much larger portion of the viral genome sequence than the qPCR assay result. This shows the higher specificity of respiratory virus detection RNA-seq, which is also not subject to the limitations of primer cross-reactivity. Lastly, we were able to strain-type viruses based on small numbers of reads, a distinction difficult to achieve using qPCR-based methods, and that could have important disease implications.

The dual RNA-seq method applied to host-dominated samples allowed us to determine that a clinically “silent” carriage of respiratory viruses results in dramatic alterations of the airway molecular phenotype with 8126 genes significantly affected. Comparison of acute in vitro experimental viral infection of airway epithelium and GO analyses of viral infection categories revealed high similarity between signatures of viral carriage without illness and active viral response. We did not observe significant differences in airway expression between asthmatic and healthy control subjects carrying a respiratory virus. We did note, however, that three of the nine modules associated with viral carriage were also associated with asthma status in uninfected subjects. Interestingly, we found that genes associated with type 2 airway inflammation, a common airway endotype in asthma, were also expressed much lower in virus-infected asthmatic and healthy children than in subjects without any viral reads detected. The association of viral infection with dampened type 2 inflammation may be due to anti-viral immune cells infiltrating the airways. Supporting this, we found viral gene response modules strongly characteristic of activated macrophage and neutrophil populations. This finding is significant, as it strongly suggests that not only are epithelial cells responding to the presence of a virus, but that the local immune system is being directed by the level of virus carriage we detect. Additionally, we identified a subset of the differentially expressed genes that were significantly altered even in subjects with very few viral reads present. These genes represented host suppression of ribosomal subunit genes by activated *EIF2AK2*, leading to inhibition of viral replication, and infiltration of airway epithelium by activated neutrophils.

We acknowledge multiple factors in our study that limit interpretation of our results and raise important questions beyond the scope of this work. Namely, mild respiratory illness may not have been reported or recognized in some subjects due to community recruitment. Additionally, symptoms and severity of rhinitis were not explicitly scored on study subjects, preventing tests of association here. Additionally, the cross-sectional nature of our study does not allow us to determine if the viral carriage we detect pre-dates, post-dates, or is unrelated to a clinically significant viral infection. It is unlikely that our detection post-dates infection, since a recent respiratory illness (6 weeks prior) was among the study exclusion criteria for the asthmatics. We acknowledge that the lack of recruitment of symptomatic patients to the study prevented us from conducting a comprehensive comparison of the effects of the viral infection without illness to viral infection effects in patients with illness. Moreover, the collection of only a single sample from subjects did not allow us to determine the persistence of this viral carriage. Furthermore, the polyA-enrichment protocol limits this method to detection of viruses with polyadenylated genomes and transcripts and would miss any viruses without those characteristics potentially present in the samples. Finally, while dual RNA-seq was initially proposed for studying host-microbiome interactions in bacterial infections [37], the approach used here would not be applicable for studying bacterial pathogens, since most bacteria do not have polyadenylated transcripts. Our results indicate that virus carriage, despite absence of illness, dramatically modulates the airway endotype of asthmatic subjects, in particular for genes related to asthma disease and asthmatic airway inflammation. These questions merit further investigation, especially considering the strong association and contribution of rhinitis severity to asthma severity in children receiving guidelines-based care [46]. This method, applied to an appropriately large sample size, would also allow for investigation of whether particular virus species and strains have gene- and pathway-specific effects on host expression. Similarly, this method will help investigate how the expression levels of individual viral genes might affect specific host genes and pathways.

## Conclusions

We applied a protocol and analytical workflow for dual RNA-seq of nasal airway epithelial brushings to concurrently detect respiratory viruses and generate the host airway transcriptome in one sample preparation and sequencing experiment. The viral detection with this method achieved 86% sensitivity when compared with qPCR-based respiratory virus detection. The sensitivity, specificity, and scope of this method resulted in the detection of multiple respiratory viruses and provided sufficient resolution to distinguish between virus species and strains in infected subjects with low total viral read percentages. Co-generation of the host transcriptome data allowed us to determine that molecular function of the airway is greatly altered by a “silent” virus carriage, which is characterized by immune cell infiltration of the airway, downregulation of ciliated cell gene expression, and modulation of type 2 inflammatory and other asthma-associated gene expression patterns.

## Methods

### GALA study subjects

All nasal brushing analysis subjects included in this study were recruited as a part of the Genes-environments and Admixture in Latino Americans II (GALA II) childhood asthma cohort. The asthmatics and controls included in this study were randomly selected. Asthma was defined by physician diagnosis and the presence of at least two asthma symptoms (coughing, wheezing, or shortness of breath). Study recruitment excluded subjects with respiratory illness as judged by a recruiting nurse evaluating cold symptoms. Additional study exclusion for asthmatics included a reported respiratory illness in the past 6 weeks. Clinical characteristics including medication usage and asthma symptoms in the past 2 weeks are listed in Additional file 1: Table S1 for study subjects.

### Processing of nasal brushings to extract RNA

Cytology brushes were used to collect airway epithelium from the posterior surface of the inferior turbinate from children with and without asthma (controls) as part of the GALA II study [47] (Fig. 2a, Additional file 1: Table S1). The anatomical location of our sampling resulted in the collection of airway epithelial cells. Collected brushes were immediately submerged in RLT Plus lysis buffer and β-mercaptoethanol and frozen at −80 °C until extraction, so that nucleic acids could be released from all sampled cells and microorganisms (host epithelial cells, viral particles, etc.). Total RNA was extracted, followed by DNase treatment.

### qPCR screening for respiratory virus infection

Respiratory virus infection was determined by measuring the following six viral species by qPCR assay: human rhinovirus (HRV), respiratory syncytial virus (RSV), human metapneumovirus (HMPV), human parainfluenza virus 1 (HPIV1), human parainfluenza virus 2 (HPIV2), and human parainfluenza virus 3 (HPIV3). Each assay was conducted using RNA extracted from the nasal airway brushings of 161 GALA II study subjects (92 asthmatics and 69 healthy controls). Specifically, RNA was reverse transcribed using oligo-dT primers and SuperScript III Reverse Transcriptase (Invitrogen). The cDNA synthesized for each sample was used for qPCR assays (5 ng per reaction) using specific primers and probes for each viral species as previously described [25]. The qPCR amplification cycling was conducted as follows: 3 minutes at 95 °C, followed by 45 cycles of denaturation for 10 seconds at 95 °C and annealing for 1 minute at 60 °C. The Ct values for each virus were used to determine the presence (Ct <45) or absence (undetectable Ct) of that viral infection within each donor.

### Viral biomarker qPCR screening for putative respiratory virus infections

Expression of the *CCL8* and *CXCL11* genes was measured by qPCR assays in RNA extracted from the nasal airway brushings of the same cohort of 161 GALA II study subjects (92 asthmatics and 69 healthy controls) as used for the virus detection qPCR assays. The cDNA was synthesized as described above, and each qPCR reaction was run using 5 ng of cDNA template and custom PrimeTime qPCR assays for the biomarkers *CCL8* (Hs.PT.58.39289889.g), *CXCL11* (Hs.PT.58.26723814), and the housekeeping gene *GusB* (Hs.PT.51.2648420) (IDT Technologies). The qPCR amplification cycling was conducted as follows: 3 minutes at 95 °C, followed by 40 cycles of denaturing for 10 seconds at 95 °C, and annealing for 30 seconds at 60 °C. The Ct values for *CCL8* and *CXCL11* were normalized to *GusB* expression levels within each sample, and gene expression values were determined using the comparative Ct analysis method [48].

### Viral pathogens detection in Ion Proton RNA-seq data

Kapa Biosystems mRNA-seq library kits (catalog number KK8421) were used to generate sequencing libraries for Ion Torrent whole transcriptome sequencing for the 21 samples with respiratory virus detected by qPCR, 2 samples with suspected viral infection based on high *CCL8* and *CXCL11* expression, and 25 randomly selected samples with no suspected asymptomatic virus carriage. Barcodes and adapters compatible with the Ion Torrent Proton sequencing instrument were used. Sequencing was conducted with P1 chips. The viral pathogen detection pipeline is schematically illustrated in Fig. 2b. Raw sequencing reads were mapped to the reference human genome hg19 (GrCHR37) with the Genomic Short-read Nucleotide Alignment Program (GSNAP) [49], and unmapped reads from this step were mapped again to the human genome with SNAP [50] using an edit distance of 14. Reads that did not map to the human genome with either algorithm and were at least 50 nucleotides long were then queried against the NCBI Nucleotide database with BLAST+ [51]. Reads of viral origin identified in this step were then assembled into longer contigs using Velvet [52]. At this step, the estimated coverage of pathogen was used as a parameter for Velvet, and was calculated as the number of viral reads, multiplied by the mean read length divided by the size of the viral genome with the highest number of BLAST hits. Assembled contigs were then queried again against the BLAST nucleotide database to verify species of viral origin. The sequence for the most common BLAST contig hit was downloaded from NCBI and indexed with SNAP. All the viral reads were mapped to this sequence with SNAP using an edit distance of 30, and final coverage depth was estimated with the BEDTtools suite [53]. The final virus coverage plot was generated with input generated by BEDTools using the ggplot2 (version 1.0.0) [54] R package in the R statistical environment. If less than 95% of the viral reads mapped to the identified closest NCBI reference sequence, and there were >50 such unmapped reads, another iteration of the virus detection pipeline was executed, starting from assembly of the unmapped reads from this step into contigs.

### Host differential expression analysis

Reads uniquely mapping as human with GSNAP [49] were used for quantification of host expression using the human hg19 iGenomes GTF file with htseq-count script [55]. For subjects with more than 12 million raw sequencing reads mapped to human genes, we performed downsampling of the reads to a maximum of 12 million reads per sample (Additional file 1: Table S2). Differential gene expression analysis between Virus-High and No-Virus subjects, as well as between Virus-Low and No-Virus subjects, was performed in R using the DESeq2 package (version 1.8.1) [56] while adjusting for asthma status. All 8126 genes significantly differentially expressed between the Virus-High and No-Virus subjects were used to build a co-expression network with the WGCNA R package (version 1.51) [30]. Gene lists forming each co-expressed module were analyzed with the DAVID functional enrichment tool [57], DAVID R Web Service (version 1.6.0) [58] and QIAGEN’s Ingenuity® Pathway Analysis (IPA®, QIAGEN, Redwood City, https://www.qiagenbioinformatics.com/products/ingenuity-pathway-analysis/). Enrichment for type 2 inflammation genes was performed with hypergeometric testing using a list of 70 genes from Poole et al., 2014 [32]. Enrichment for activated and non-activated immune cell types was performed using the Human Immune Cell Transcriptome dataset (GSE3982) [31] obtained from the NCBI Gene Expression Omnibus (GEO) with the Generally Applicable Gene-set Enrichment (GAGE) R package (version 2.18.0) [59] and Gene Set Enrichment Analysis (GSEA) [60], in the same way as previously described [61]. All other statistical analyses and plots were generated in the R environment using the following packages: edgeR (version 3.10.2) [62] for generating MDS plots, and ggplot2 (version 2.1.0) [54] for generating coverage plots of viral genomes. Reported correlations with viral depth were calculated using Spearman correlations. Collapsed gene expression for the 100 Virus-Low differentially expressed genes was calculated with the collapseRows function [63] implemented in the WGCNA R package.

### In vitro HRV stimulation

Human tracheal airway epithelial cells from three donors were cultured using a modified Schlegel method as previously described [64–67]. Basal cells were seeded and grown at an air-liquid interface as described previously [68]. Briefly, 6.5-mm inserts (Corning, catalog number 3470) were coated with 2.7 mg/ml collagen (PureCol Bovine Collagen #5005-B, Advanced Biomatrix, San Diego, CA, USA). Epithelial cells were seeded in airway media 1 [68] at 1.2 × 10^5^ cells per insert. At 24 hours post-plating, airway media 1 was replaced with airway medium 2 containing 2% Ultroser G [68]. Cultures were air-lifted upon the appearance of confluent cell monolayers 72 hours after cells were seeded, and maintained in airway media 2 for 21 days for cell differentiation. After 21 days of growth at the air-liquid interface, paired differentiated cells from each donor were mock-infected (control) or infected with human rhinovirus A16 (HRV-A16). Both apical and basolateral chambers of the inserts were washed with phosphate-buffered saline (PBS), and fresh airway media 2 was added to the basolateral chamber. Apical chambers were infected with HRV-A16 at a multiplicity of infection (MOI) of 5 in X-Vivo 10 serum-free media (Lonza) for 24 hours in a 37 °C humidified incubator containing 5% CO_2_; control inserts were incubated with X-Vivo media alone in the apical chamber. Both the apical and basolateral chambers of the inserts were washed three times with warmed PBS, fresh airway media 2 containing USG was added to the basolateral chamber, and the apical chamber was left at the air-liquid interface for an additional 24 hours in a 37 °C incubator. Inserts were washed with PBS, cell monolayers lysed in RLT buffer, and RNA was extracted using an RNA Miniprep Kit (QIAGEN).

RNA-seq libraries were prepared using the standard protocol for the Ion AmpliSeq Transcriptome Human Gene Expression Kit. The prepared libraries were multiplexed and sequenced on the Ion Torrent Proton sequencer. Raw sequencing reads were mapped to human transcriptome reference (hg19) and gene count tables were generated with ampliseqRNA plugin v.5.0.0.0 and Torrent Suite v5.0.4 (Additional file 1: Table S5). Differential expression analysis was performed with the DESeq2 R package (version 1.4.5) [56], including information on sample pairing in the applied model. We used 483 of 493 genes (ones with matching gene IDs between the two studies) with adjusted *P* values <0.01, and absolute log2 expression fold changes > =1 were then used to assess whether viral infections without illness in vivo demonstrate similar expression changes as in vitro airway epithelial HRV infection.

### Hematoxylin and eosin (H&E) staining of nasal airway brushing cytospins

A second nasal brushing collected at the same time as the brushing used for RNA-seq was solubilized, and the cells were cytospun, H&E stained, and imaged by standard methods.

## Additional files

Additional file 1:
**Table S1.** Clinical characteristics of subjects. **Table S2.** Gene count table for 48 RNA-seq nasal brushing subjects from GALA cohort. **Table S3.** Virus-High DESeq differential expression analysis. **Table S4.** DAVID enrichment for Virus High DEGs. **Table S5.** Gene count table for HRV in vitro stimulation dataset. **Table S6.** HRV in vitro DEGs. **Table S7.** Virus Low Differential Expression Analysis. **Table S8.** WGCNA gene2module. **Table S9.** DAVId for modules. **Table S10.** immune cell enrichments for modules. (XLS 18739 kb)
Additional file 2: Figure S1. Study design. **Figure S2.** Genome coverage for viruses detected in Ion Torrent Proton sequenced samples. **Figure S3.** Comparison of in vivo and in vitro log2 gene expression fold changes by infection status. **Figure S4.** WGCNA gene correlation dendrogram showing nine detected gene co-expression modules. **Figure S5.** H&E staining of nasal airway epithelial brushing cell cytospin from a viral-infected subject. (PDF 30208 kb)
