# Correction to: Dual RNA-seq reveals viral infections in asthmatic children without respiratory illness which are associated with changes in the airway transcriptome

**DOI:** 10.1186/s13059-018-1423-3

**Published:** 2018-04-10

**Authors:** Agata Wesolowska-Andersen, Jamie L. Everman, Rebecca Davidson, Cydney Rios, Rachelle Herrin, Celeste Eng, William J. Janssen, Andrew H. Liu, Sam S. Oh, Rajesh Kumar, Tasha E. Fingerlin, Jose Rodriguez-Santana, Esteban G. Burchard, Max A. Seibold

**Affiliations:** 1 Center for Genes, Environment, and Health, National Jewish Health, Denver, CO USA; 2 Department of Medicine, University of California, San Francisco, CA USA; 3 Department of Medicine, National Jewish Health, Denver, CO USA; 4 Department of Pediatrics, National Jewish Health, 1400 Jackson St, Denver, CO 80206 USA; 5 Children’s Hospital Colorado and University of Colorado School of Medicine, Aurora, CO USA; 6 Department of Pediatrics, The Ann and Robert H. Lurie Children’s Hospital of Chicago, Northwestern University Feinberg School of Medicine, Chicago, IL USA; 7 Department of Biomedical Research, National Jewish Health, Denver, CO USA; 8 Centro de Neumologia Pediatrica, San Juan, Puerto Rico; 9 Department of Bioengineering and Therapeutic Sciences, University of California, San Francisco, CA USA; 10 Division of Pulmonary Sciences and Critical Care Medicine, University of Colorado School of Medicine, Aurora, CO USA

## Correction

In our recent article [1], it has come to our attention that the sample labels are not consistent between Table 1, the data labels deposited in the Sequence Read Archive, and Additional file 1: Table S2. We are therefore providing an updated Additional file 1: Table S2 so identical samples now have the same label.

## Additional file

Additional file 1: **Table S2.** Gene count table for 48 RNA-seq nasal brushing subjects from GALA cohort. (XLSX 5732 kb)
