# Supplementary material for: Dual RNA-seq reveals viral infections in asthmatic children without respiratory illness which are associated with changes in the airway transcriptome
Source: Genome Biol. 2017 Jan 19;18:12. doi: 10.1186/s13059-016-1140-8 (PMC5244706; doi:10.1186/s13059-016-1140-8)

Figure S1. Study design. This figure provides brief descriptions of the datasets used and shows the flow of analyses conducted in this study. Blue boxes show different datasets, green boxes represent conducted analyses, and red boxes represent results obtained at different analysis steps of this study. Abbreviations used: qPCR = quantitative polymerase chain reaction, DEGs = differentially expressed genes, WGCNA = weighted gene co-expression network analysis.

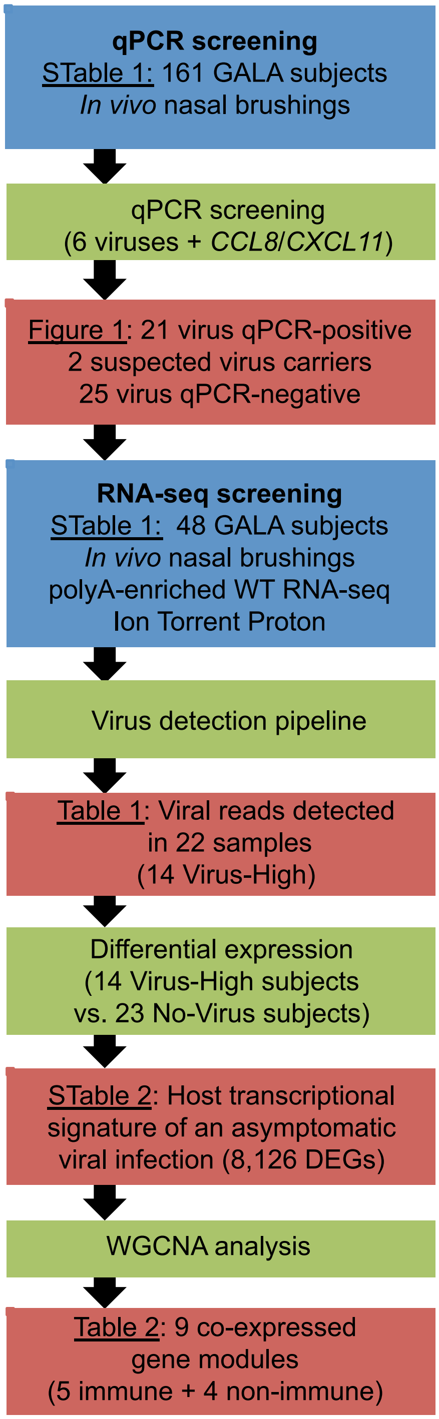

Figure S2. Genome coverage for viruses detected in Ion Torrent Proton sequenced samples. Viral read pileups are shown along the detected viral genomes for each viral-carrying sample with >20 viral reads. Horizontal black lines represent the position of the assembled contigs obtained with Velvet, plotted at their reported coverage depth. The viral transcript locations are shown below the read pileups.

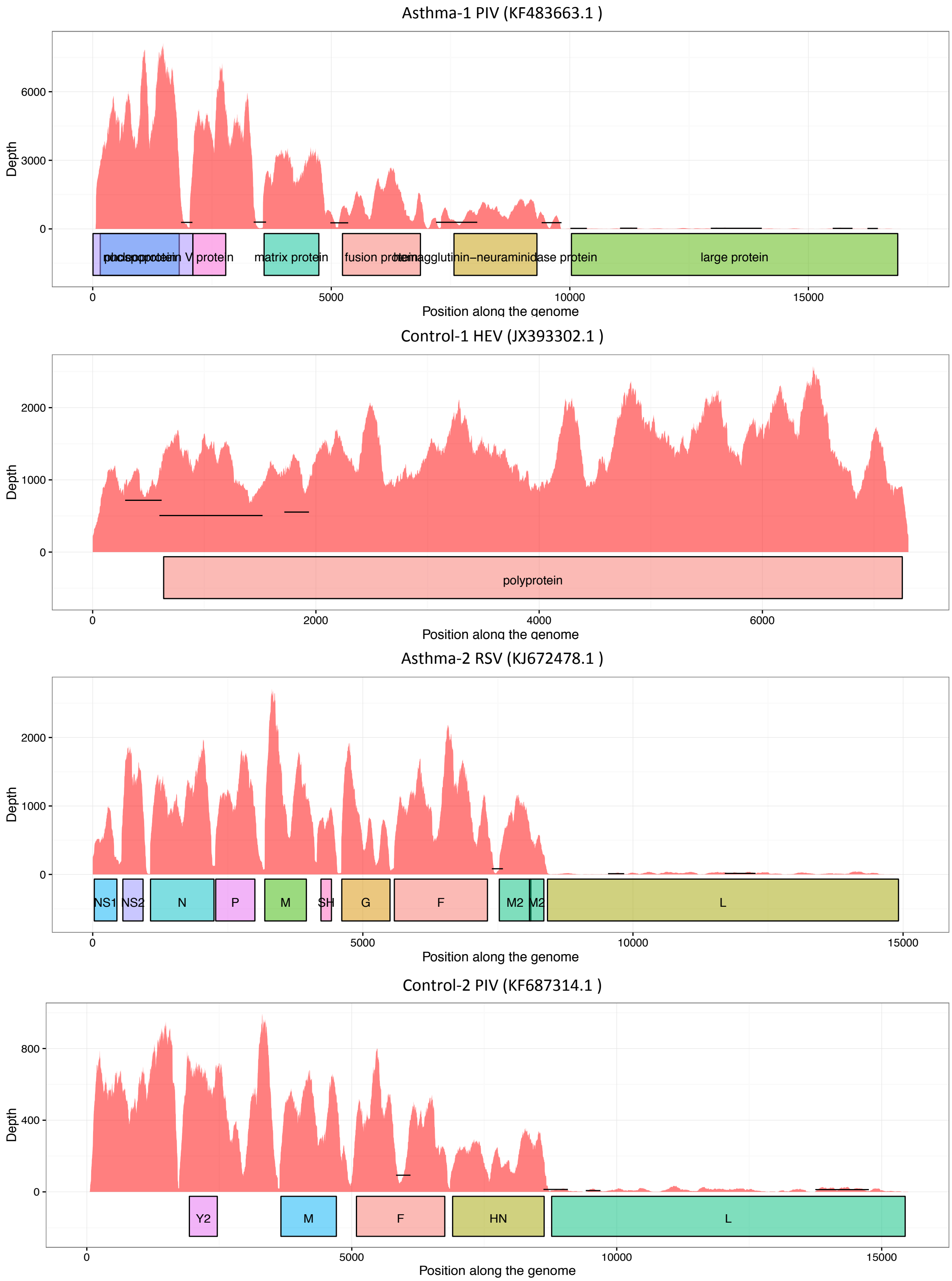

Asthma-1 PIV (KF483663.1)
Depth
6000
3000
0
phosphoprotein
nucleoprotein
V protein
matrix protein
fusion protein
hemagglutinin–neuraminidase protein
large protein
0
5000
10000
15000
Position along the genome
Control-1 HEV (JX393302.1)
Depth
2000
1000
0
polyprotein
0
2000
4000
6000
Position along the genome
Asthma-2 RSV (KJ672478.1)
Depth
2000
1000
0
NS1
NS2
N
P
M
SH
G
F
M2
M2
L
0
5000
10000
15000
Position along the genome
Control-2 PIV (KF687314.1)
Depth
800
400
0
Y2
M
F
HN
L
0
5000
10000
15000
Position along the genome

Control-3 HRV (DQ473511.1)

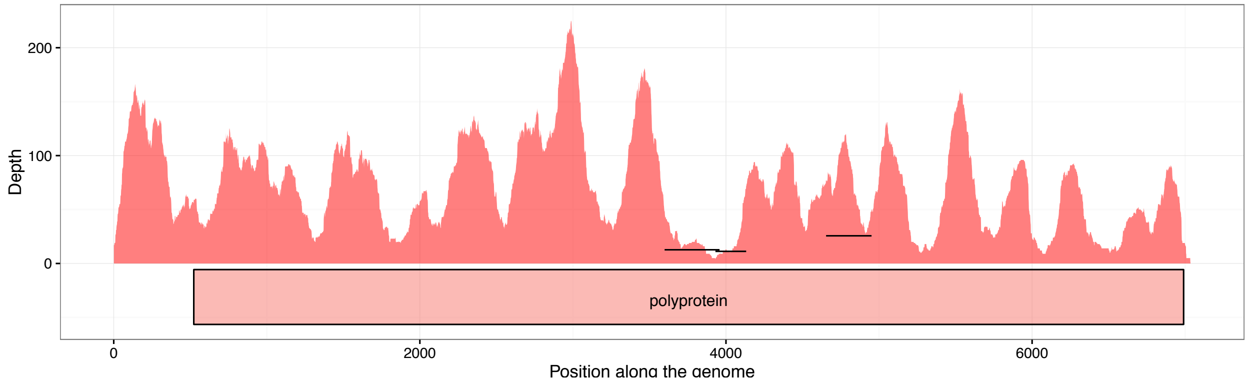

Asthma-3 PIV (KF530212.1)

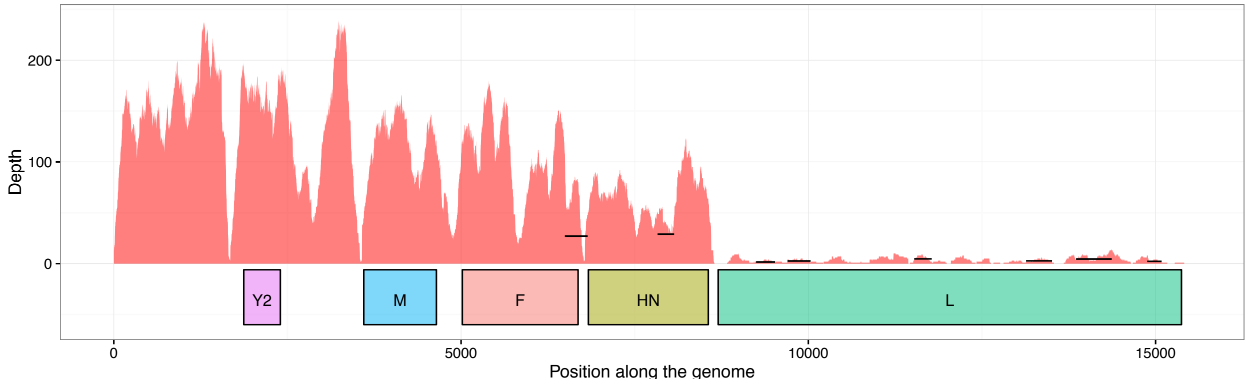

Control-4 HRV (JN541266.1)

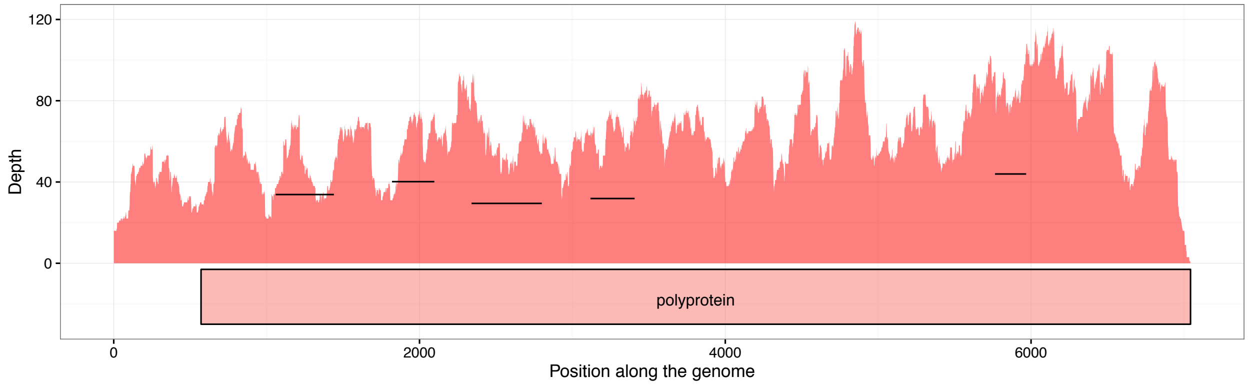

Asthma-4 HRV (JF317015.1)

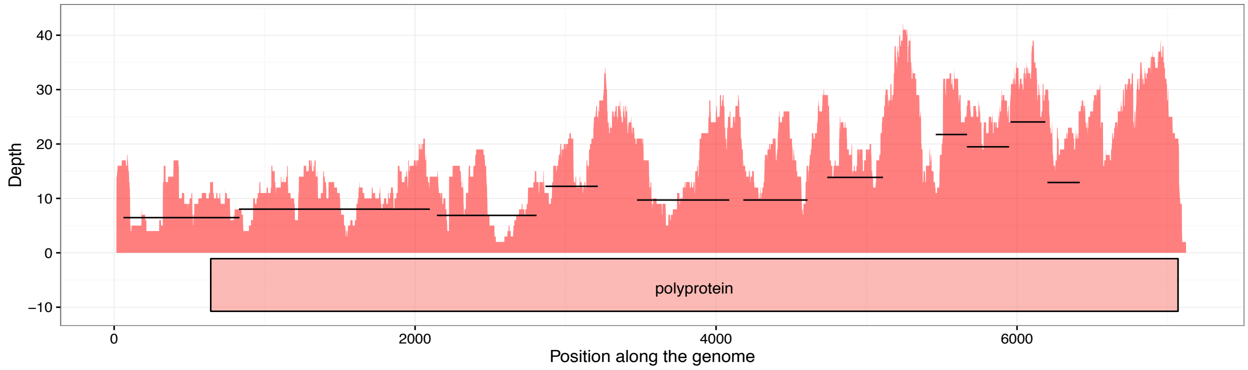

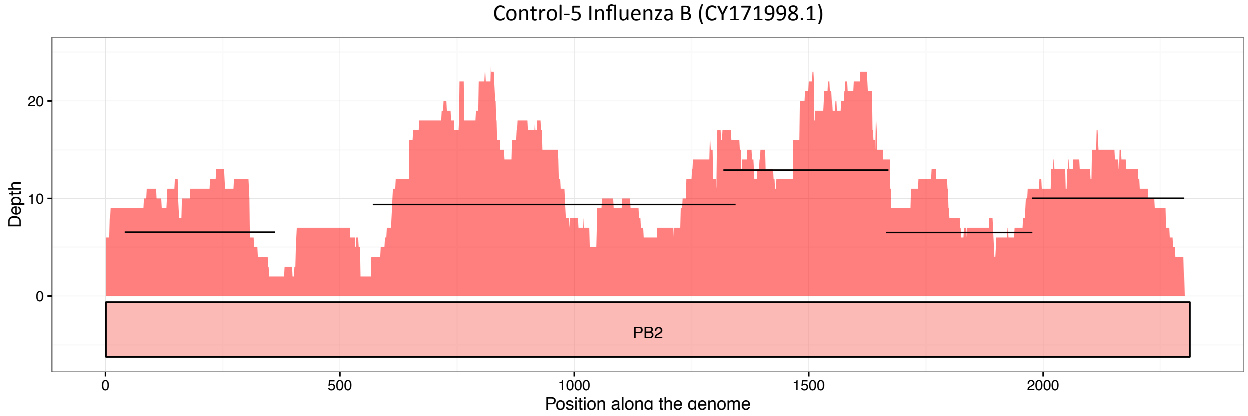
Control-5 Influenza B (CY171998.1)
Depth
20
10
0
PB2
0
500
1000
1500
2000
Position along the genome

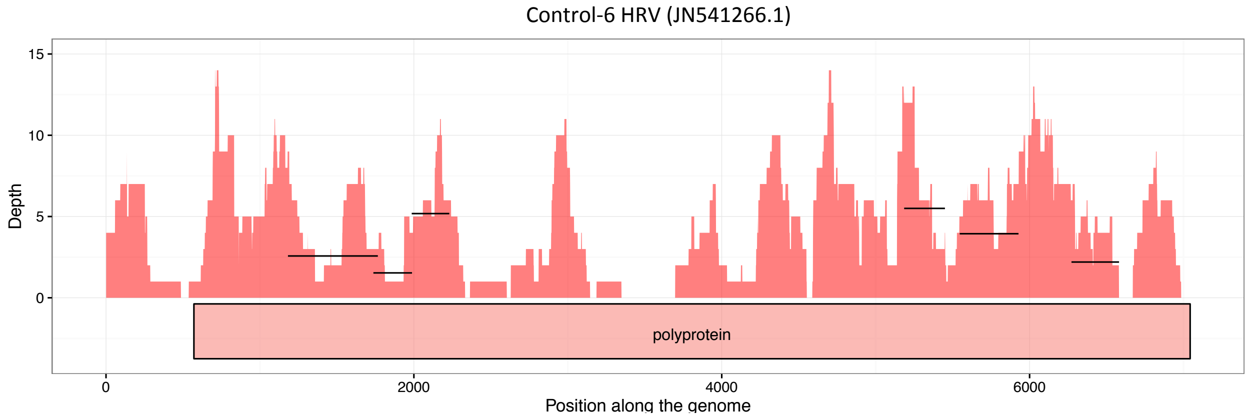
Control-6 HRV (JN541266.1)
Depth
15
10
5
0
polyprotein
0
2000
4000
6000
Position along the genome

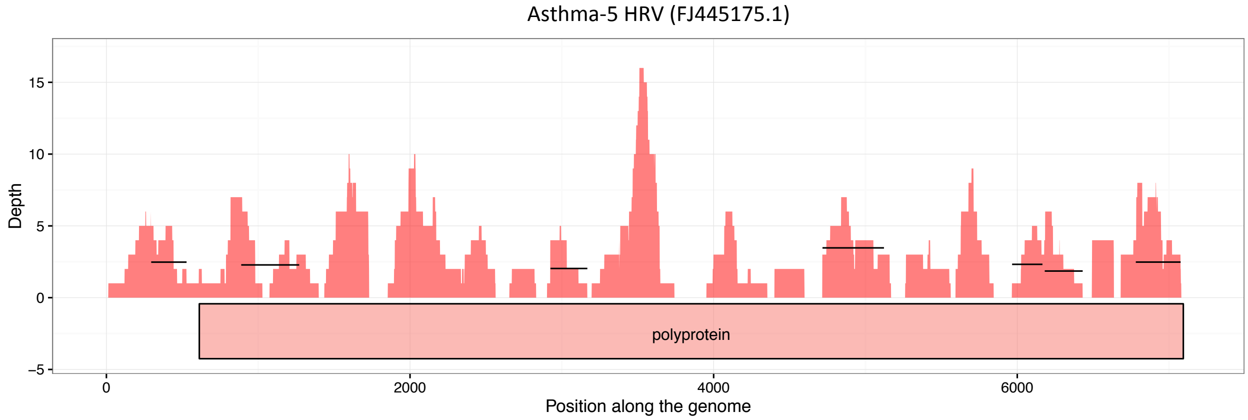
Asthma-5 HRV (FJ445175.1)
Depth
15
10
5
0
−5
polyprotein
0
2000
4000
6000
Position along the genome

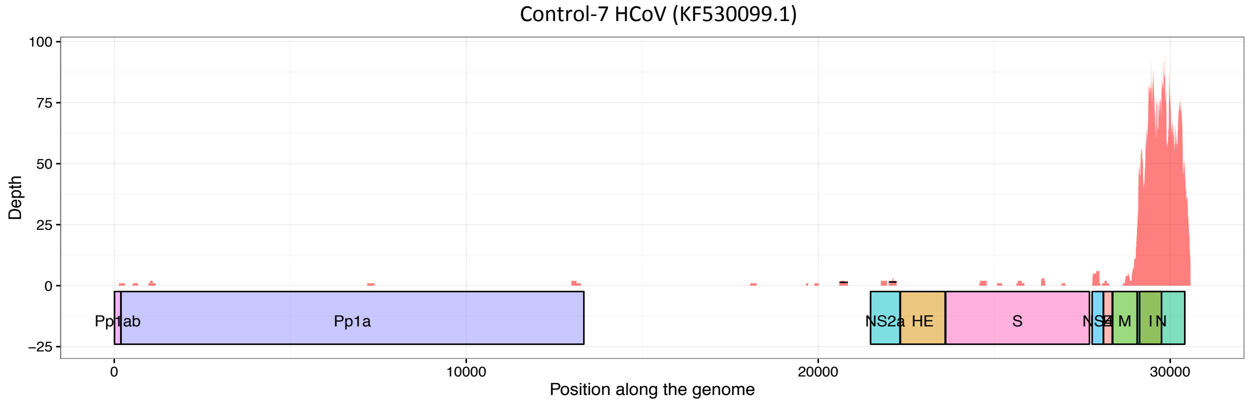
Control-7 HCoV (KF530099.1)
Depth
100
75
50
25
0
−25
Pp1ab
Pp1a
NS2a
HE
S
M
N
0
10000
20000
30000
Position along the genome

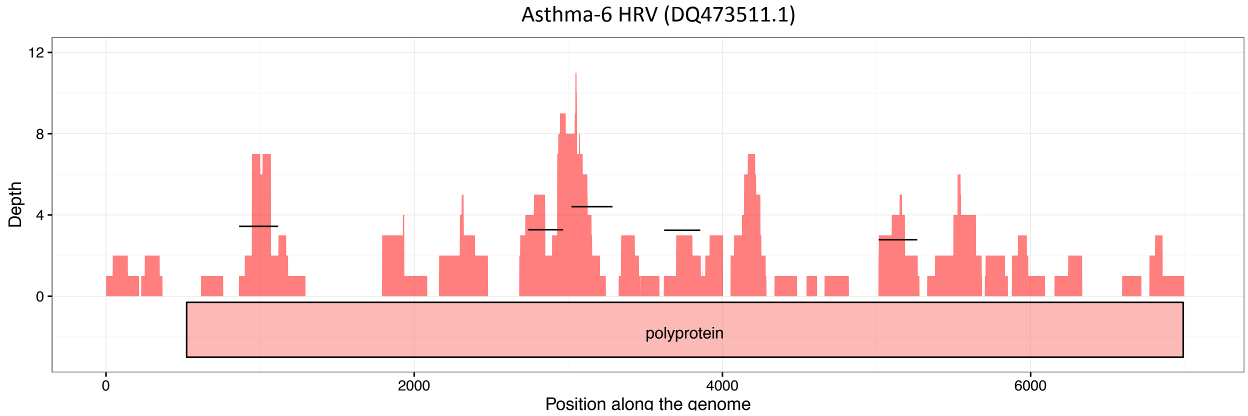
Asthma-6 HRV (DQ473511.1)
Depth
Position along the genome
polyprotein
0
4
8
12
2000
4000
6000

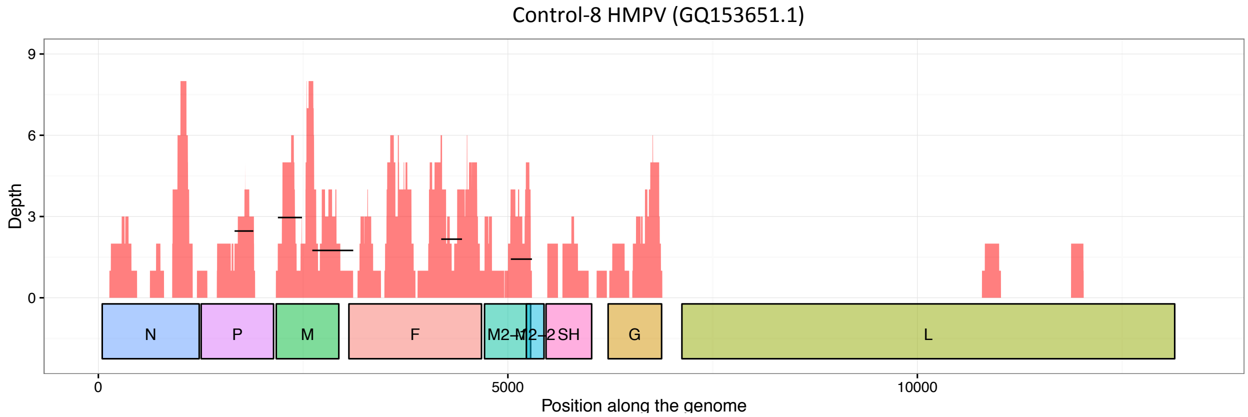
Control-8 HMPV (GQ153651.1)
Depth
Position along the genome
N
P
M
F
M2-1
M2-2
SH
G
L
0
3
6
9
5000
10000

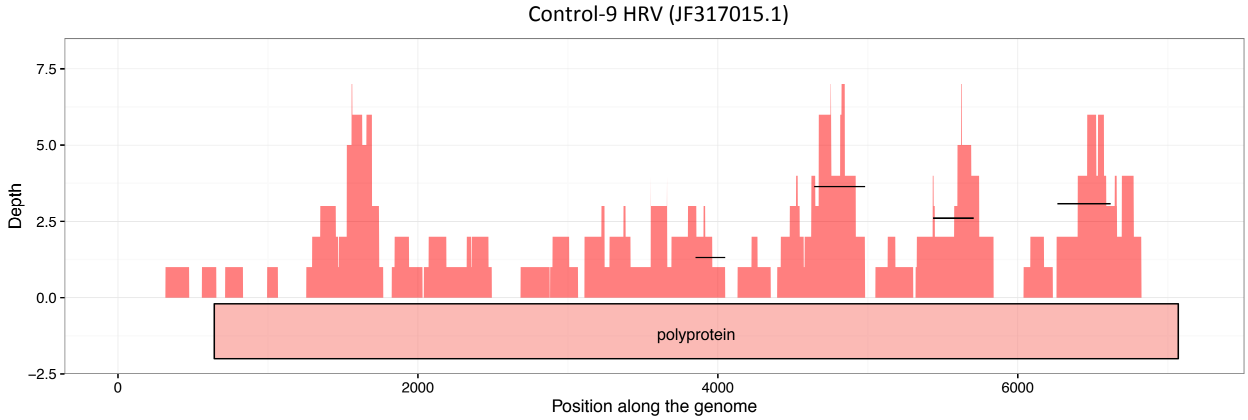
Control-9 HRV (JF317015.1)
Depth
Position along the genome
polyprotein
−2.5
0.0
2.5
5.0
7.5
2000
4000
6000

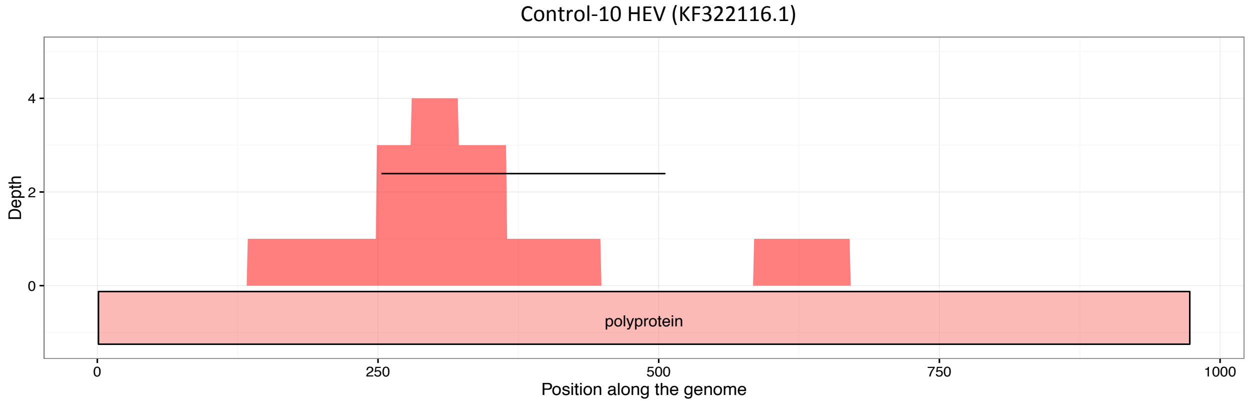
Control-10 HEV (KF322116.1)
Depth
Position along the genome
polyprotein
0
2
4
250
500
750
1000

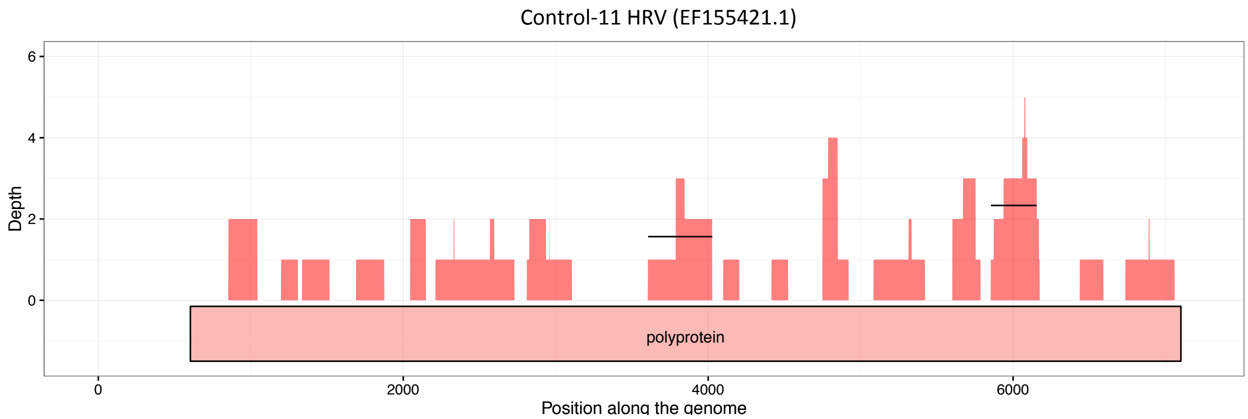
Control-11 HRV (EF155421.1)
Depth
6
4
2
0
polyprotein
0
2000
4000
6000
Position along the genome

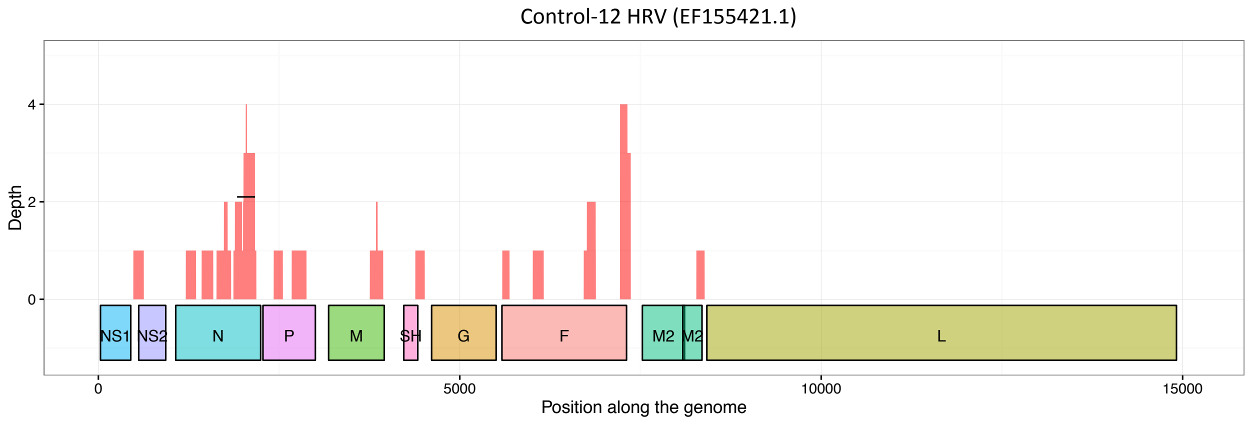
Control-12 HRV (EF155421.1)
Depth
4
2
0
NS1
NS2
N
P
M
SH
G
F
M2
M2
L
0
5000
10000
15000
Position along the genome

Figure S3. Comparison of *in vivo* and *in vitro* log2 gene expression fold changes by infection status. Genes marked in red indicate 199 genes significantly upregulated in response to virus in the *in vivo*, but not in the *in vitro* samples.

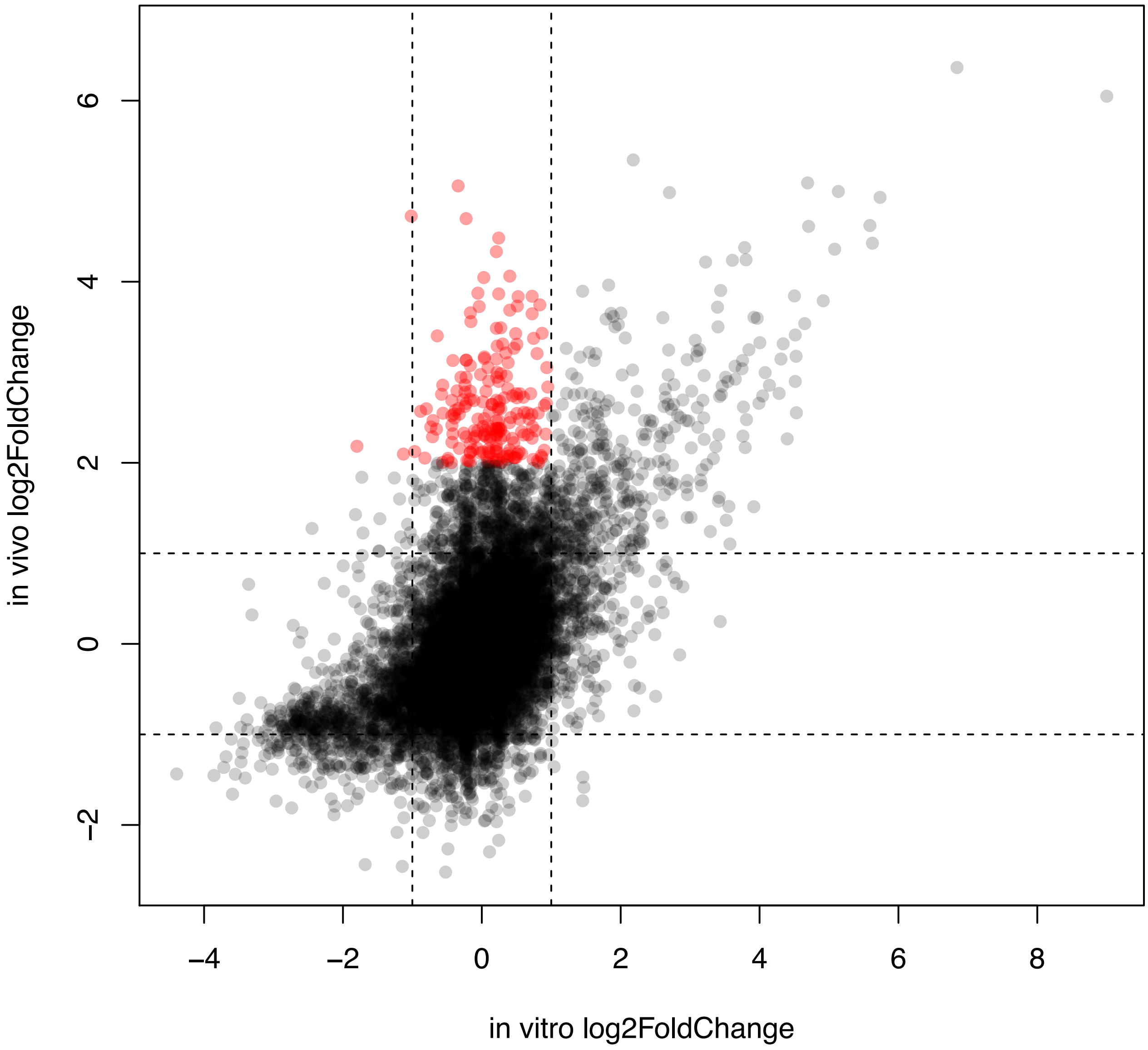

Figure S4. WGCNA gene correlation dendrogram showing nine detected gene co-expression modules. Dendrogram represents the strength of gene correlations, and the colored bars below show the location of detected gene co-expression modules. Grey denoted genes not assigned to any co-expression module.

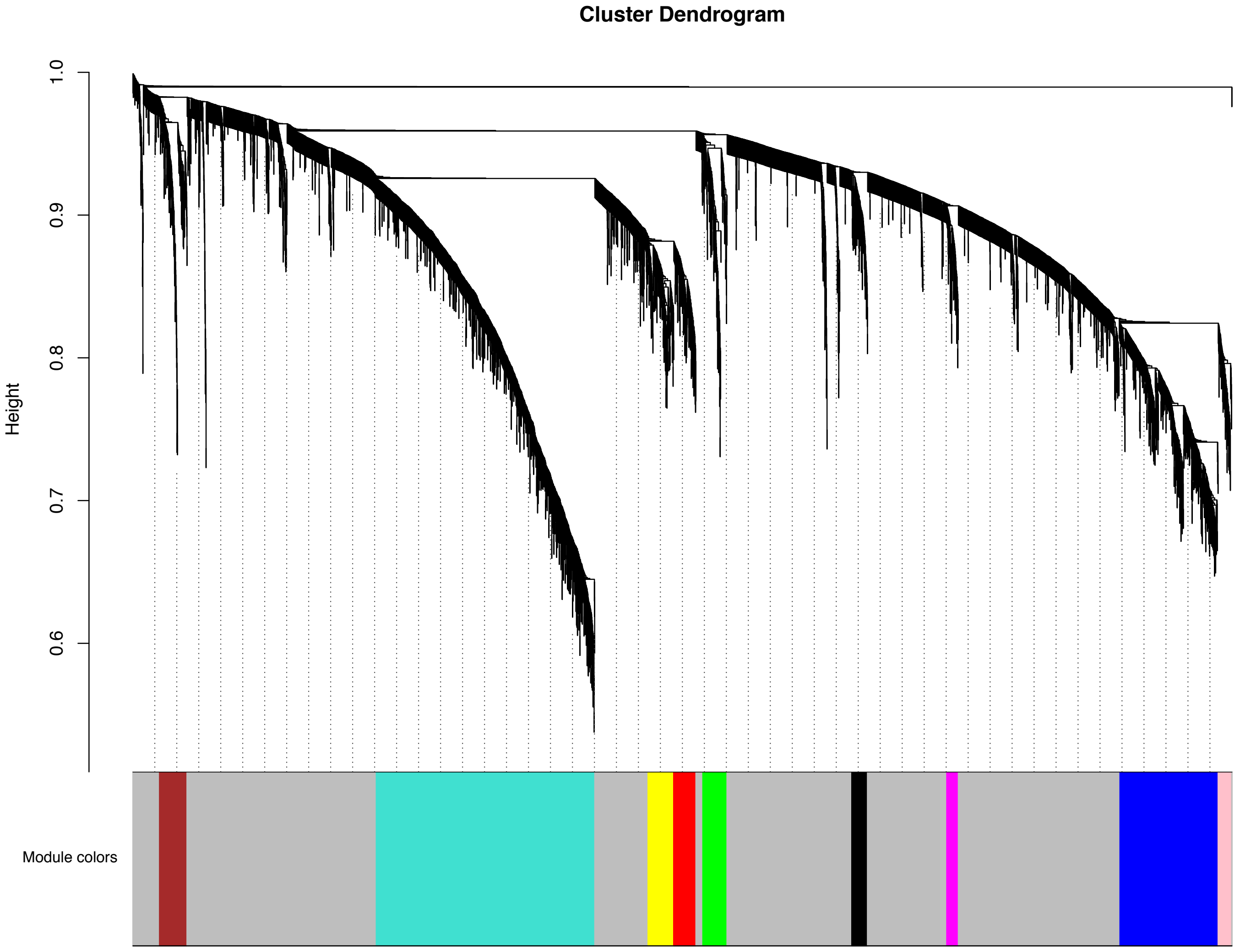

Figure S5. H & E staining of nasal airway epithelial brushing cell cytospin from a viral-infected subject.

Red, blue, and black arrows point to macrophages, a neutrophil, and epithelial cells, respectively. Images at 40X magnification.

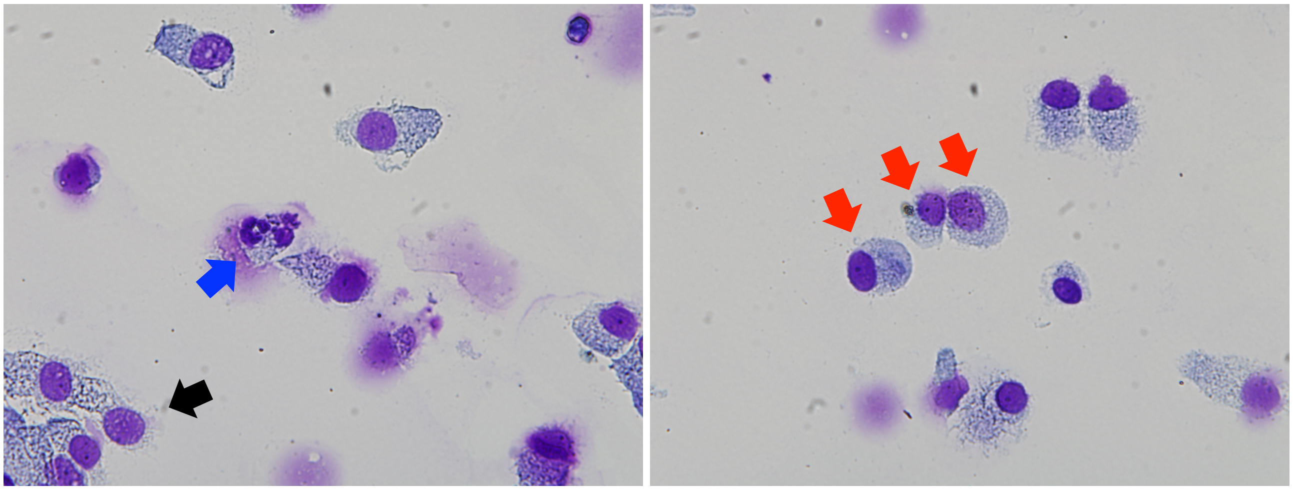

Supplement: Additional file 2: Figure S1. — Study design. Figure S2. Genome coverage for viruses detected in Ion Torrent Proton sequenced samples. Figure S3. Comparison of in vivo and in vitro log2 gene expression fold changes by infection status. Figure S4. WGCNA gene correlation dendrogram showing nine detected gene co-expression modules. Figure S5. H&E staining of nasal airway epithelial brushing cell cytospin from a viral-infected subject. (PDF 30208 kb) [file 13059_2016_1140_MOESM2_ESM.pdf]
